# 3-Carboxylic Acid and Formyl-Derived Coumarins as Photoinitiators in Photo-Oxidation or Photo-Reduction Processes for Photopolymerization upon Visible Light: Photocomposite Synthesis and 3D Printing Applications

**DOI:** 10.3390/molecules26061753

**Published:** 2021-03-21

**Authors:** Mahmoud Rahal, Bernadette Graff, Joumana Toufaily, Tayssir Hamieh, Guillaume Noirbent, Didier Gigmes, Frédéric Dumur, Jacques Lalevée

**Affiliations:** 1 CNRS, IS2M UMR 7361, Université de Haute-Alsace, F-68100 Mulhouse, France; mahmoud-rahal@outlook.com (M.R.); bernadette.graff@uha.fr (B.G.); 2 Université de Strasbourg, 67081 Strasbourg, France; 3 Laboratory of Materials, Catalysis, Environment and Analytical Methods (MCEMA) and LEADDER Laboratory, Faculty of Sciences, Doctoral School of Sciences and Technology (EDST), Lebanese University, Beirut 6573-14, Lebanon; joumana.toufaily@ul.edu.lb (J.T.); tayssir.hamieh@ul.edu.lb (T.H.); 4 SATIE-IFSTTAR, Université Gustave Eiffel, Campus de Marne-La-Vallée, 25, allée des Marronniers, F-78000 Versailles, France; 5 CNRS, ICR UMR 7273, Aix Marseille Université, F-13397 Marseille, France; guillaume.noirbent@outlook.fr (G.N.); didier.gigmes@univ-amu.fr (D.G.)

**Keywords:** coumarin, free radical polymerization, LED, photocomposites, direct laser write

## Abstract

In this paper, nine organic compounds based on the coumarin scaffold and different substituents were synthesized and used as high-performance photoinitiators for free radical photopolymerization (FRP) of meth(acrylate) functions under visible light irradiation using LED at 405 nm. In fact, these compounds showed a very high initiation capacity and very good polymerization profiles (both high rate of polymerization (Rp) and final conversion (FC)) using two and three-component photoinitiating systems based on coum/iodonium salt (0.1%/1% *w*/*w*) and coum/iodonium salt/amine (0.1%/1%/1% *w*/*w*/*w*), respectively. To demonstrate the efficiency of the initiation of photopolymerization, several techniques were used to study the photophysical and photochemical properties of coumarins, such as: UV-visible absorption spectroscopy, steady-state photolysis, real-time FTIR, and cyclic voltammetry. On the other hand, these compounds were also tested in direct laser write experiments (3D printing). The synthesis of photocomposites based on glass fiber or carbon fiber using an LED conveyor at 385 nm (0.7 W/cm^2^) was also examined.

## 1. Introduction

The development of new low-cost, environmentally friendly, and energy-efficient polymer synthesis remains more than ever at the heart of academic and industrial concerns and the subject of many new research strategies. In fact, thanks to technological development, light sources which are at the same time inexpensive, efficient, and with low energy consumption have been developed recently to induce photopolymerization reactions [1,2,3,4]. Nowadays, photopolymers are present in several fields such as coatings [5], dentistry [6], automotive [7], cosmetics [8], 3D printing, and holography [9], etc. For most of these industrial fields, photochemical polymerization uses ultraviolet radiation, a technique widely known as UV curing. However, this pathway based on UV lamps (Hg lamps) remains energy-consuming. Moreover, the ultraviolet light is harmful to human health (carcinogenic) and characterized by particularly low light penetration, which is a challenge for the photopolymerization of thick and filled samples [10]. Therefore, alternatives to UV lamps and the use of longer wavelengths (near UV or visible) can be advantageous. The use of light-emitting diodes (LEDs) perfectly fit this requirement for safer/cheaper, and more efficient irradiation devices than UV lamps or UV lasers [11,12,13,14]. In parallel, it is important to develop new photoinitiating systems able to absorb in the near UV or the visible range where their absorption spectrum overlaps that of the LED emission. To obtain this type of system, it is necessary to develop new organic molecules carrying chromophore groups capable of shifting their absorption spectrum towards the near-UV-visible range. These molecules will be called photoinitiator (PI), which can absorb the light and generate reactive species (in combination with additives) able to initiate the photopolymerization process.

**Scheme 1:**
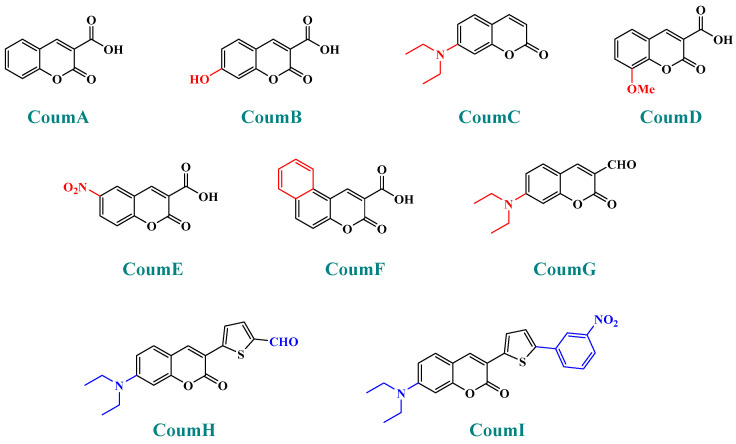
The new series of coumarins (CoumA–CoumI) examined as photoinitiators of polymerization.

In this paper, nine coumarin derivatives (noted Coum in Scheme 1) varying by the substitution pattern at the 3- and 7-positions of the coumarin core were synthesized and evaluated as photoinitiators for the FRP of acrylate and methacrylate monomers. In fact, coumarin derivatives have already been tested as photoinitiators of FRP and they have shown good polymerization profile (Rp and FC) as well as good photochemical and photophysical properties [15,16,17,18,19]. 

However, in the present work, coumarin-3-carboxylic acids, coumarin-3-aldehydes varying by the substitution pattern of the coumarin core and a coumarin of extended aromaticity have been studied as photoinitiators. Comparisons of the three families of coumarins have revealed that the substitution of the 3-position by electron-withdrawing groups such as a formyl group could improve the reactivity. The presence of a strong electron-donating group at the 7-position, such as diethylamine or a naphthalene group, could reinforce the electronic delocalization and the photoinitiating ability of the different systems. An optimum situation was found when electron-donating and electron-accepting groups were attached at both extremities of the coumarin core. Considering that the nitro group is among the most electron-withdrawing group, a coumarin bearing this electron acceptor was also designed and synthesized. 

In fact, coumarin derivatives are usually characterized by very high fluorescence emission and can be used as fluorescent chromophores for several applications [20]. They are also characterized by high molar extinction coefficients in the near-UV and the visible range [19]. These novel coumarin-based photoinitiators were tested in photopolymerization of acrylate functions (TMPTA or TA) in both Thick (1.4 mm) and Thin sample (25 µm) using two and three-component photoinitiating systems PISs based on Coum/Iodonium salt (0.1%/1% *w*/*w*) and Coum/Iodonium salt/amine (NPG) (0.1%/1%/1% *w*/*w*/*w*). These systems were also used in 3D printing and photocomposite synthesis. These dyes are characterized by very high extinction coefficients with a broad absorption extending over the near UV/visible and high quantum yields were determined by fluorescence quenching. It is important to note that coumarin shows a dual photo-oxidation and photo-reduction character.

## 2. Results

Photoinitiation ability, the performance of photopolymerization, photophysical and photochemical properties as well as chemical mechanisms associated with the photopolymerization processes will be discussed in detail.

### 2.1. Synthesis of the Different Dyes

**Scheme 2:**
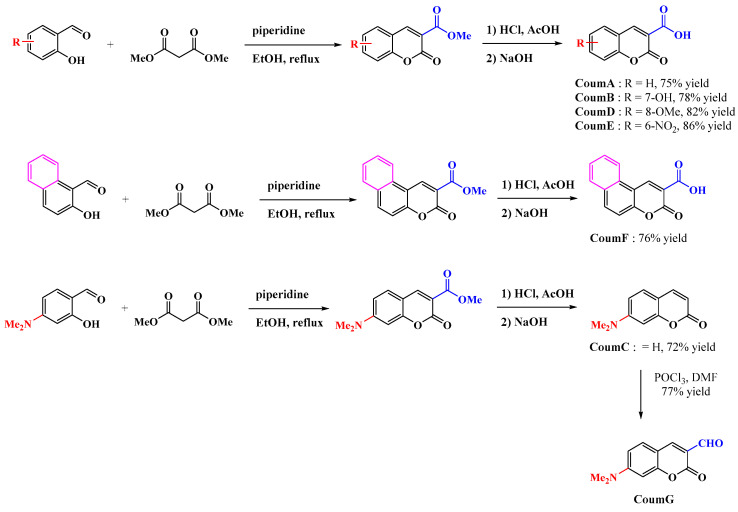
Synthetic routes to CoumA–CoumG.

As mentioned in the introduction section, three families of coumarins have been examined as photoinitiators of polymerization. The first family concerned coumarin-3-carboxylic acids. The five dyes were prepared in solution by condensation of diethyl malonate with *ortho*-hydroxyarylaldehydes [21]. After hydrolysis of esters in acidic conditions (a mixture of hydrochloric acid and acetic acid), the solution was neutralized to provide the different dyes with reaction yields ranging from 75% for CoumA to 86% yield for CoumE. A similar procedure was used for CoumC except that the hydrolysis of the intermediate ester coumarin resulted in a decarboxylation reaction, providing CoumC in 72% yield. The presence of the dimethylamino group in CoumC is essential to activate the decarboxylation reaction since this reaction was not observed for the other coumarins, maintaining the acidic function on the coumarins (See Scheme 2) [22]. Using the Vilsmeier Haack reaction, CoumC could be converted as CoumG in a 77% yield.

**Scheme 3:**
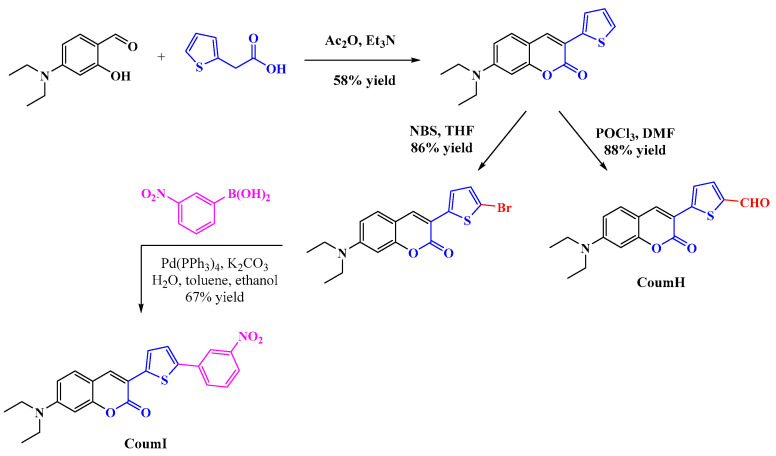
Synthetic routes to CoumH and CoumI.

Finally, CoumH and CoumI could be prepared starting from 2-thiopheneacetic acid and 4-diethylamino-2-hydroxybenzaldehyde. By Knoevenagel reaction, 7-(diethylamino)-3-(thiophen-2-yl)-2*H*-chromen-2-one could be obtained in 58% yield and by means of a Vilsmeier Haack reaction, CoumH was isolated in pure form in 88% yield. Conversely, CoumI was prepared in two steps, first by bromination of 7-(diethylamino)-3-(thiophen-2-yl)-2*H*-chromen-2-one in 86% yield, followed by a Suzuki cross-coupling reaction with 3-nitrophenylboronic acid. Using this procedure, CoumI was obtained in 67% yield (See Scheme 3).

### 2.2. Light Absorption Properties

**Figure 1:**
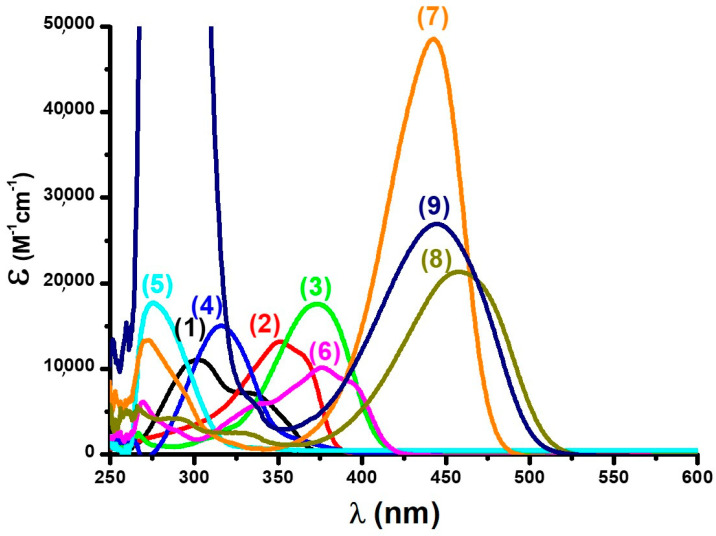
UV-visible absorption spectra of the investigated compounds based on coumarin derivatives in ACN: (**1**) CoumA, (**2**) CoumB, (**3**) CoumC, (**4**) CoumD, (**5**) CoumE, (**6**) CoumF, (**7**) CoumG, (**8**) CoumH, and (**9**) CoumI.

UV-visible absorption spectra of the different coumarins in acetonitrile are depicted in Figure 1 (See also Table 1). These organic compounds are characterized by a high molar extinction coefficient in both near-UV and visible range (e.g., CoumC **ε** ~ 18000 M^−1^cm^−1^ at 374 nm and 3500 M^-1^cm^−1^ at 405 nm, and CoumF **ε** ~ 10200 M^−1^cm^−1^ at 376 nm and 4800 M^−1^cm^−1^ at 405 nm). So, these absorption properties afford a good overlap with the emission spectrum of the LEDs used in this work (LED at 405 nm for FRP, LED at 375 nm for the photolysis experiments and LED at 385 nm for the photocomposites synthesis).

**Figure 2:**
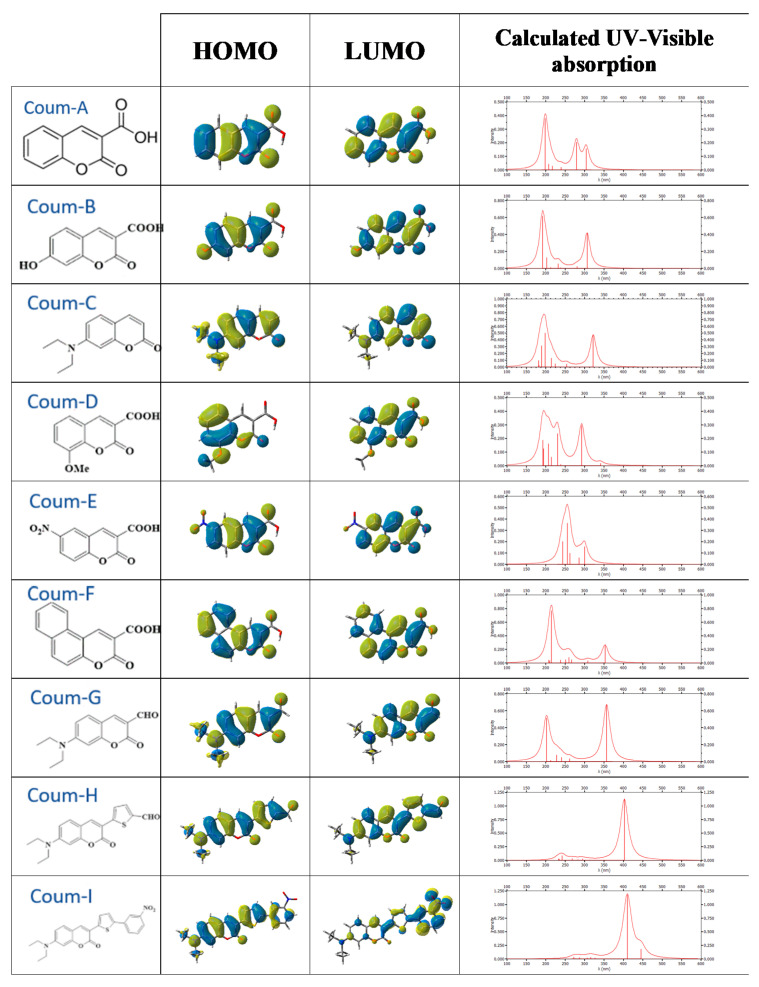
HOMO and LUMO frontier orbitals and their respective calculated UV-visible absorption spectra for the different investigated compounds at the UB3LYP/6-31G* level.

In fact, the presence of different substituents on the coumarin scaffold can affect the absorption properties (Figure 2) of these compounds and their molar extinction coefficients can be affected. For example, taking CoumA as a standard structure among these 10 compounds, we observed a shift towards higher absorption range (e.g., CoumB, CoumD, and CoumF are strongly shifted), and towards lower absorption range (e.g., CoumE), so a bathochromic effect is observed by introduction of electron donor group (such as OH, OMe, and NR_2_) and a hypsochromic effect is observed by introduction of electron acceptor group (e.g., NO_2_ in case of CoumE).

The electron-donating effect of these substituents is presented by ascending order: CoumH ˃ CoumI ˃ CoumG ˃ CoumF ˃ CoumC ˃ CoumD ˃ CoumB.

### 2.3. Free Radical Photopolymerization

#### 2.3.1. Photopolymerization of Methacrylate Function of Mix-MA

**Figure 3:**
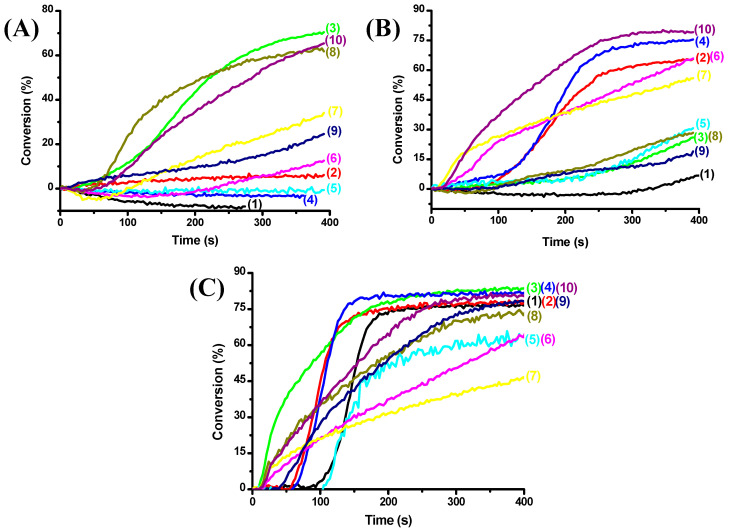
Photopolymerization profiles of methacrylate functions (conversion vs. irradiation time) using MIX-MA in thick sample (1.4 mm) under visible light irradiation using a LED at 405 nm: (**A**) Coum/Iod (0.1%/1% *w*/*w*), (**B**) Coum/NPG (0.1%/1% *w*/*w*) and **(C)** Coum/Iod/NPG (0.1%/1%/1% *w*/*w*/*w*): (1) CoumA, (2) CoumB, (3) CoumC, (4) CoumD, (5) CoumE, (6) CoumF, (7) CoumG, (8) CoumH, (9) CoumI and (10) Iod/NPG (1%/1% *w*/*w*). Irradiation starts at t = 10 s.

The FRP profiles of methacrylate functions using Mix-MA as the benchmark monomer was performed in thick sample and in the presence of two or three-component PISs based on Coum/Iod (or NPG) (0.1%/1% *w*/*w*) or Coum/Iod/NPG (0.1%/1%/1% *w*/*w*/*w*) respectively, upon visible light irradiation with a LED at 405nm are given in Figure 3 (See also Table 2).

The obtained results show that Coum/Iod (or NPG) is less reactive than three-component PISs (Coum/Iod/NPG), this result can be explained by a higher yield of reactive species (radicals) in the presence of Iod/NPG which is not able, alone, to initiate the FRP (e.g., FC = 24% for CoumI/Iod vs. 76% for CoumI/Iod/NPG, and FC = 0% for CoumA/Iod vs. 76% for CoumA/Iod/NPG; show Figure 3A,C curve 1). In fact, CoumB, CoumD, CoumF and CoumG showed a photoreduction process rather than a photo-oxidation process (e.g., FC = 0% for CoumB/Iod vs. 67% for CoumB/NPG, and FC = 0% for CoumD/Iod vs. 75% for CoumD/NPG), but CoumC and Coum8 show an opposite behavior with a photo-oxidation process probably more favorable than the photoreduction (FC = 70% for CoumC/Iod vs. 28% for CoumC/NPG; Figure 3A,B curve 3). The FRP profiles also show a low rate of polymerization, this can be due to the high oxygen inhibition effect.

#### 2.3.2. Photopolymerization of Acrylates (TMPTA or TA)

In fact, iodonium salt or NPG alone cannot initiate the FRP of acrylate at 405 nm due to their absorption in the UV range [17,23]. Therefore, the coumarins derivatives are introduced in order to improve the absorption properties of photosensitive formulations.

**Figure 4:**
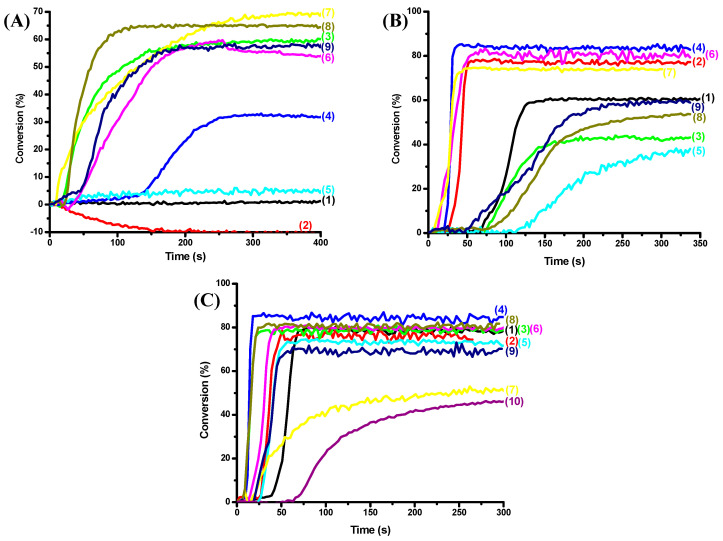
Photopolymerization profiles of acrylate functions (conversion vs irradiation time) using TMPTA in thick sample (1.4 mm) under visible light irradiation using a LED at 405 nm: (**A**) Coum/Iod (0.1%/1% *w*/*w*), (**B**) Coum/NPG (0.1%/1% *w*/*w*) and (**C**) Coum/Iod/NPG (0.1%/1%/1% *w*/*w*/*w*): (1) CoumA, (2) CoumB, (3) CoumC, (4) CoumD, (5) CoumE, (6) CoumF, (7) CoumG, (8) CoumH, (9) CoumI and (10) Iod/NPG (1%/1% *w*/*w*). The molar concentrations for 0.1 % *w*/*w* are 0.0055, 0.0051, 0.0049, 0.0048, 0.0045, 0.0044, 0.0043, 0.0032, and 0.0025 M for CoumA, CoumB, CoumC, CoumD, CoumE, CoumF, CoumG, CoumH, CoumI, respectelievly. The irradiation starts at t = 10 s.

Firstly, the most of Coumarin derivatives show high extinction coefficients at 405 nm. The photopolymerization profiles of acrylate functions in thick (1.4 mm) or thin (25 µm) samples (conversion vs. irradiation time) using TMPTA (or TA) as benchmark monomers are depicted in Figure 4 (see also Table 2 and Table 3). The obtained results show that the two-component PISs based on Coum/Iod (0.1%/1% *w*/*w*) (or Coum/NPG) are able to strongly initiate the FRP, but a very higher performance [Final conversion (FC) and polymerization rate (Rp)] was acquired using the three-component PISs based on Coum/Iod/NPG which is quite efficient in the FRP of acrylate functions upon LED at 405 nm (e.g., FC = 60% for CoumC/Iod (0.1%/1% *w*/*w*) vs. 80% for CoumC/Iod/NPG (0.1%/1%/1% *w*/*w*/*w*), Figure 4A and B curve 3).

Moreover, the Iod/NPG (1%/1% *w*/*w*) couple weakly initiates the FRP (FC = 47%). This is ascribed to the formation of a charge transfer complex (CTC) between Iod and NPG [24] which is able to generate reactive species when it absorbs light. Clearly, the presence of Coumarin as photoinitiator is improving the performance of the photopolymerization processes.

Some of the coumarins can show both photoreduction (electron transfer from NPG to Coumarin) and photoxidation (electron transfer from Coumarin to Iod) processes, while other derivatives show only photoreduction process, such as CoumA, CoumB and CoumE (e.g., FC = 0% for CoumB/Iod (0.1%/1% *w*/*w*) vs. FC = 78% for CoumB/NPG (0.1%/1% *w*/*w*)).

### 2.4. D Printing Experiments Using Coum/Iod/amine PISs and Optical Microscopy Characterization

**Figure 5:**
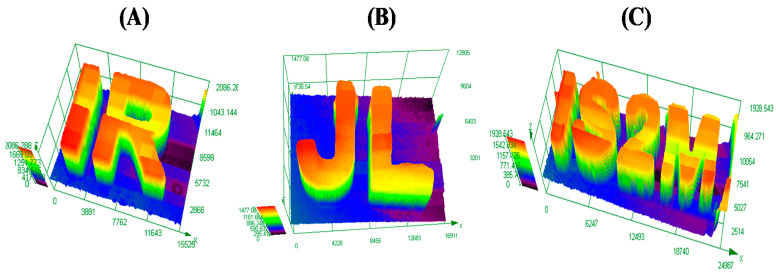
Characterization of 3D patterns by numerical optical microscopy obtained by free radical photopolymerization experiment (using TA or TMPTA as benchmark monomer) using a diode laser at 405 nm: (**A**) CoumC/Iod/4,N-N-TMA (0.05%/0.5%/0.235% *w*/*w*/*w*) in TA, (**B**) CoumH/Iod/TMA (0.05%/0.5%/0.19% *w*/*w*/*w*) in TMPTA and **(C)** CoumD/Iod/4,N-N-TMA (0.05%/0.5%/0.275% *w*/*w*/*w*) in TMPTA.

New 3D patterns were obtained by direct laser write experiments of Coum/Iod/amine PISs using a laser diode at 405 nm and characterized by optical microscopy. These 3D patterns were obtained under air using different PISs based on Coum/Iod/TMA (0.05%/0.5%/0.235% *w*/*w*/*w*) in TA or TMPTA (See Figure 5). In fact, the high photosensitivity of this resin allowed an efficient polymerization process in the irradiated area so a high spatial resolution is observed. Markedly, a great thickness is obtained (~2090 µm) and these patterns were carried out in a very short irradiation time (~2–3 min). Using a well-established Type I photoinitiator (diphenyl(2,4,6-trimethylbenzoyl)phosphine oxide—TPO) in similar direct laser write conditions; similar performances can be reached but requiring a higher content (0.5% *w*/*w*). This latter result demonstrates the interest in using Coum derivatives. It is important to note that the 3D patterns based on CoumC exhibit a blue fluorescence when these structures are characterized by the light of the microscope.

### 2.5. Near-UV Conveyor Experiments for the Synthesis of Photocomposites Using Coum/Iod/NPG (0.1%/1%/1% w/w/w)

Generally, photocomposites are materials composed of at least two components: matrix and reinforcement. The mixture of these two components leads to new interesting properties that the two components separately do not have. The production of composites in the last decades and until today represents a very dynamic market in different fields such as aeronautics, automotive, wind power, and buildings. So, due to their very high mechanical resistance and chemical resistance, the glass fibers are used in this work as a matrix for the photocomposite synthesis.

**Figure 6:**
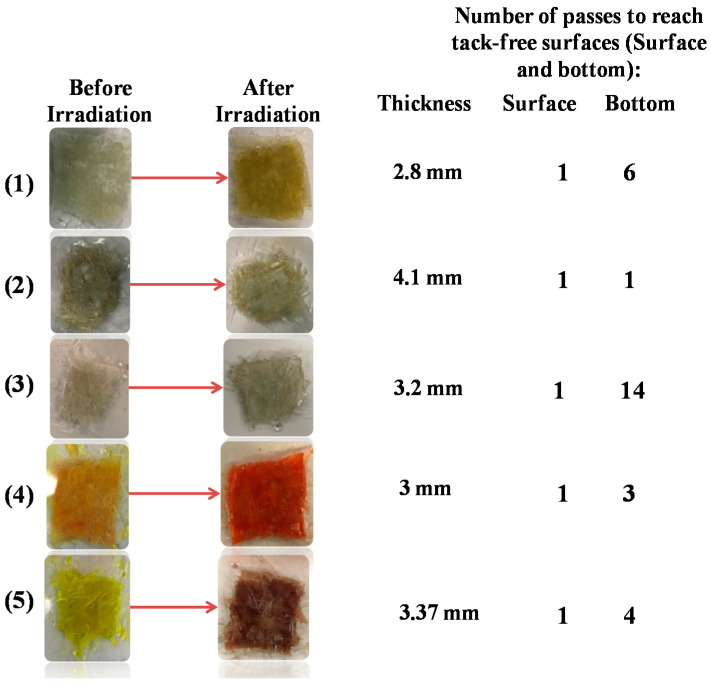
Photocomposites manufactured upon near-UV irradiation at 385 nm (0.7 W/cm^2^) using glass fiber/resin (50%/50% *w*/*w*) in the presence of three-component PISs based on Coum/Iod/NPG (0.1%/1%/1% *w*/*w*/*w*): (**1**) CoumC, (**2**) CoumD, (**3**) CoumF, (**4**) CoumG, and (**5**) CoumH.

In this work, the proposed coumarin derivatives were tested for access to photocomposites upon near-UV light using a LED conveyor at 385 nm (0.7 W/cm^2^). The curing results obtained are summarized in Figure 6. Firstly, photocomposites were prepared by impregnation of glass fibers with an acrylic resin (TMPTA) (50% glass fibers/50% acrylic resin) and irradiated upon a LED at 385 nm. Remarkably, a very fast polymerization was observed using Coum/Iod/NPG (0.1%/1%/1% *w*/*w*/*w*), where both the surface and the bottom are tack-free after some passes.

## 3. Discussion

**Scheme 4:**
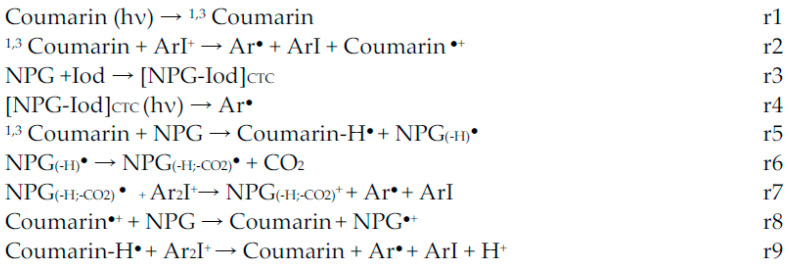
Proposed chemical mechanisms.

For a better understanding of the photoinitiation ability, the photochemical properties of the studied coumarins were investigated. More particularly, their photolysis behaviors, fluorescence quenchings, and redox properties were investigated in the presence of additives (amine/iodonium salt), allowing to establish the photochemical mechanisms (see Scheme 4 below). 

### 3.1. Steady-State Photolysis of Coumarins

**Figure 7:**
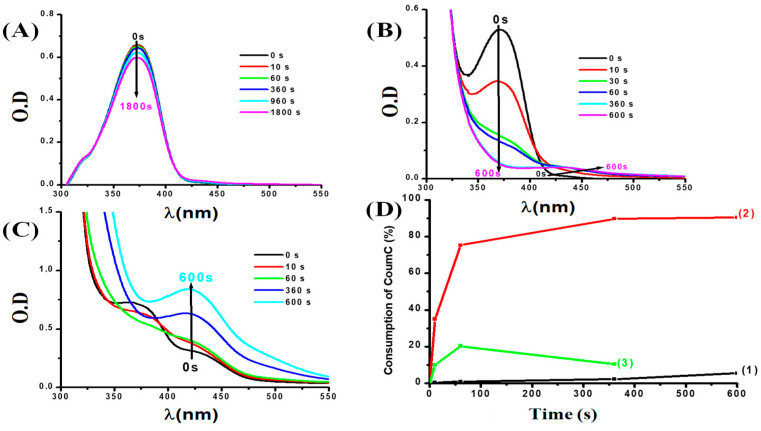
(**A**) Photolysis of CoumC alone in ACN, (**B**) photolysis of CoumC with Iod (10^−2^ M) in ACN, (**C**) photolysis of CoumC with Iod/NPG (10^−2^M) couple, (**D**) percentage of consumption of CoumC (1) without Iod, and with (2) with Iod (10^−2^ M), and (3) with Iod/NPG vs. irradiation time—upon exposure to the LED@375 nm in ACN.

Steady-state photolysis of coumarins derivatives in ACN and under irradiation light using a LED at 375 nm have been performed to explain the obtained results in FRP. So, the photolysis of one of these compounds (CoumC) is presented in Figure 7. First of all, the photolysis of CoumC alone upon irradiation at 375 nm is very slow compared to that obtained with Iod, which is very fast. In fact, the appearance of a weak peak between 425 and 500 nm and the evolution of the absorption peak of CoumC shows that a high interaction between CoumC and Iod took place by an electron transfer process, this process induced, during the irradiation, a photolysis of the CoumC and generation of new photoproducts. On the other hand, the photolysis of CoumC with Iod/NPG couple was very slow (Figure 7D curve 3) and poor consumption was obtained; these results can be explained by a high regeneration of CoumC in three-component PISs.

### 3.2. Fluorescence Quenching and Cyclic Voltammetry Experiments for the Coumarins

**Figure 8:**
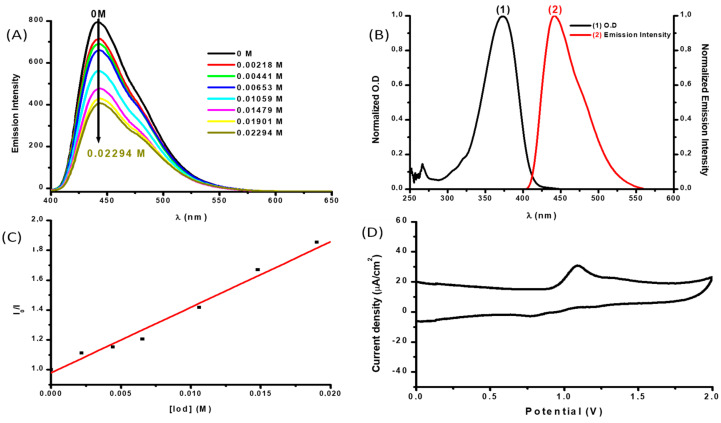
(**A**) Fluorescence quenching of CoumC by Iod, (**B**) E_S1_ determination, (**C**) determination of K_SV_ (Stern–Volmer coefficient), and (**D**) oxidation potential (E_ox_) determination of CoumC.

Fluorescence quenching and emission spectra of the different coumarins (e.g., CoumC) have been carried out in ACN and reported in Figure 8. Firstly, where the emission intensity of CoumC decreases when we added Iod or NPG, so an interaction between ^1^Coum-C and Iod (or NPG) occurs, this result is in full agreement with FRP and photolysis experiments shown above. To compare the reactivity of different coumarin with Iod or NPG, the Stern-Volmer coefficient (Ksv) have been calculated according to Equation (1). For example, a very high quenching of CoumF with NPG and poor quenching of CoumC with NPG were observed, so Ksv for CoumF is higher than that of CoumC (Ksv = 44 M^−1^ for CoumC and 400 M^−1^ for CoumF), therefore a high electron transfer quantum yield is obtained for CoumF ϕ = 0.9) compared to that obtained for CoumC (ϕ = 0.6) (Table 4)

ϕ_S1_ = K_SV_[Iod]/(1 + K_SV_[Iod]) (1)

The free energy change (ΔG) for the electron transfer between coumarins and Iod or NPG is an important parameter to evaluate the feasibility of this process. ΔG can be extracted from the E_S1_ and the electrochemical properties (E_ox_ and E_red_) (using Equation (1)) e.g., ΔG = −2.39 eV for CoumF/Iod which is more reactive in FRP of acrylate functions (TA monomer) (FC = 86%). All these data are gathered in Table 4.

Finally, the FRP results of acrylate functions can be explained by a global mechanism based on the different results obtained by the characterization techniques (steady-state photolysis, Fluorescence quenching and cyclic voltammetry). First of all, the photoinitiator (Coumarin) goes to its excited state once it absorbs suitable light energy, and as it is not able to give reactive species alone, the Iod salt (or NPG), therefore, interacts with its excited state and will be able to dissociate and give reactive species responsible to initiate the FRP (r1–r2). The addition of NPG to the photosensitive formulation is very important because of the formation of a charge-transfer complex between Iod salt and NPG [Iod-NPG]_CTC_ able to generate reactive species (r3–r4). Moreover, a hydrogen transfer process from NPG to Coumarins can occur which generates two types of radicals (Coum-H^●^, NPG_(-H)_^●^) (r5). In fact, a decarboxylation of NPG_(-H)_^●^ can take place and leads to the radical formation (NPG_(-H, -CO2)_^●^), which react with Iod salt to produce reactive species (Ar^●^ and NPG_(-H, CO2)_^+^) (r6–r7). Ar^●^ and NPG_(-H,-CO2)_^●^_)_ (r1–r9) radicals are assumed as the reactive species responsible to the FRP of the (meth)acrylate functions. The coumarins consumption is reduced in three-component PIS (Figure 6); this can be explained by a regeneration of the photoinitiator, which is in agreement on r8-r9 (See Scheme 4). 

The photoinitiation ability is a strong interplay between these different reactions (r1-r9), but their light absorption properties and intersystem crossing behavior (singlet vs. triplet state pathways, lifetimes) must also be taken into account. Therefore, a deeper characterization of their structure/reactivity/efficiency relationship is beyond the scope of the present work.

## 4. Materials and Methods

### 4.1. Synthesis of the Coumarins

All reagents and solvents were purchased from Aldrich, Alfa Aesar, or TCI Europe and used as received without further purification. Mass spectroscopy was performed by the Spectropole of Aix-Marseille University. ESI mass spectral analyses were recorded with a 3200 QTRAP (Applied Biosystems SCIEX) mass spectrometer. The HRMS mass spectral analysis was performed with a QStar Elite (Applied Biosystems SCIEX) mass spectrometer. Elemental analyses were recorded with a Thermo Finnigan EA 1112 elemental analysis apparatus driven by the Eager 300 software. ^1^H and ^13^C NMR spectra were determined at room temperature in 5 mm o.d. tubes on a Bruker Avance 400 spectrometer and on a Bruker Avance 300 spectrometer of the Spectropole: The ^1^H chemical shifts were referenced to the solvent peak CDCl_3_ (7.26 ppm), and the ^13^C chemical shifts were referenced to the solvent peak CDCl_3_ (77 ppm). 7-(Diethylamino)-3-(thiophen-2-yl)-2*H*-chromen-2-one and 3-(5-bromothiophen-2-yl)-7-(diethylamino)-2*H*-chromen-2-one used as intermediates of reaction have been synthesized according to procedures previously reported in the literature, without modifications and in similar yields [18]. 

Synthesis of 2-oxo-2*H*-chromene-3-carboxylic acid (CoumA)


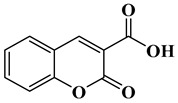


Dimethyl malonate (2.64 g, 20 mmol, M = 132.11 g/mol) and piperidine (1 mL, 10 mmol) were mixed and added to a solution of salicylaldehyde (1.22 g, 10 mmol, M = 122.12 g/mol) dissolved in absolute ethanol (30 mL). After stirring and heating to reflux for 6 h, the solvent was removed under reduced pressure. Then, concentrated HCl (20 mL) and acetic acid (20 mL) were added for hydrolysis and the solution was refluxed overnight. After cooling, the solution was poured onto ice and aq. 40% NaOH was added until pH = 5. A pale precipitate formed. After stirring for another 30 min, the mixture was filtered, washed with water, pentane, and dried under vacuum (1.43 g, 75% yield). ^1^H NMR (400 MHz, DMSO-d_6_) δ 8.75 (s, 1H), 7.91 (dd, *J* = 7.7, 1.2 Hz, 1H), 7.80–7.68 (m, 1H), 7.42 (dd, *J* = 15.4, 7.9 Hz, 2H). Analyses were consistent with those previously reported in the literature [25].

Synthesis of 7-hydroxy-2-oxo-2*H*-chromene-3-carboxylic acid (CoumB),


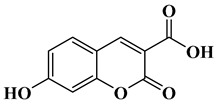


Dimethyl malonate (2.64 g, 20 mmol, M = 132.11 g/mol) and piperidine (1 mL, 10 mmol) were mixed and added to the solution of 2,4-dihydroxybenzaldehyde (1.38 g, 10 mmol, M = 138.12 g/mol) dissolved in absolute ethanol (30 mL). After stirring and heating to reflux for 6 h, the solvent was removed under reduced pressure. Then, concentrated HCl (20 mL) and acetic acid (20 mL) were added for hydrolysis, and the solution was refluxed overnight. After cooling, the solution was poured onto ice and aq. 40% NaOH was added until pH = 5. A pale precipitate formed. After stirring for another 30 min, the mixture was filtered, washed with water, pentane, and dried under vacuum (1.61 g, 78% yield). ^1^H NMR (400 MHz, DMSO-*d_6_*) δ 8.68 (s, 1H), 7.75 (d, *J* = 8.6 Hz, 1H), 6.85 (dd, *J* = 8.6, 2.3 Hz, 1H), 6.74 (d, *J* = 2.1 Hz, 1H). Analyses were consistent with those previously reported in the literature [25].

Synthesis of 7-(diethylamino)-2*H*-chromen-2-one (CoumC)


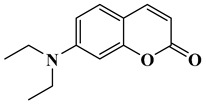


Dimethyl malonate (2.64 g, 20 mmol, M = 132.11 g/mol) and piperidine (1 mL, 10 mmol) were mixed and added to the solution of 4-(Diethylamino)-salicylaldehyde (1.93 g, 10 mmol) dissolved in absolute ethanol (30 mL). After stirring and heating to reflux for 6 h, the solvent was removed under reduced pressure. Then, concentrated HCl (20 mL) and acetic acid (20 mL) were added for hydrolysis and the solution was refluxed overnight. After cooling, the solution was poured onto ice and aq. 40% NaOH was added until pH = 5. A pale precipitate formed. After stirring for another 30 min, the mixture was filtered, washed with water, pentane, and dried under vacuum (1.56 g, 72% yield). ^1^H NMR (400 MHz, CDCl_3_) δ 7.53 (d, *J* = 9.3 Hz, 1H), 7.24 (d, *J* = 8.8 Hz, 1H), 6.60–6.44 (m, 2H), 6.03 (d, *J* = 9.3 Hz, 1H), 3.41 (q, *J* = 7.1 Hz, 4H), 1.21 (t, *J* = 7.1 Hz, 6H). Analyses were consistent with those previously reported in the literature [26].

Synthesis of 8-methoxy-2-oxo-2*H*-chromene-3-carboxylic acid (CoumD)


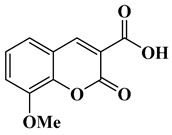


Dimethyl malonate (2.64 g, 20 mmol, M = 132.11 g/mol) and piperidine (1 mL, 10 mmol) were mixed and added to the solution of 3-methoxysalicylaldehyde (1.52 g, 10 mmol, M = 152.15 g/mol) dissolved in absolute ethanol (30 mL). After stirring and heating to reflux for 6 h, the solvent was removed under reduced pressure. Then, concentrated HCl (20 mL) and acetic acid (20 mL) were added for hydrolysis and the solution was refluxed overnight. After cooling, the solution was poured onto ice and aq. 40% NaOH was added until pH = 5. A pale precipitate formed. After stirring for another 30 min, the mixture was filtered, washed with water, pentane, and dried under vacuum (1.80 g, 82% yield). ^1^H NMR (400 MHz, DMSO-*d_6_*) δ 8.50 (s, 1H), 7.42–7.26 (m, 3H), 3.91 (s, 3H). Analyses were consistent with those previously reported in the literature [27].

Synthesis of 6-nitro-2-oxo-2*H*-chromene-3-carboxylic acid (CoumE)


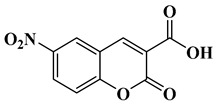


Dimethyl malonate (2.64 g, 20 mmol, M = 132.11 g/mol) and piperidine (1 mL, 10 mmol) were mixed and added to the solution of 2-hydroxy-5-nitrobenzaldehyde (1.67 g, 10 mmol, M = 167.12 g/mol) dissolved in absolute ethanol (30 mL). After stirring and heating to reflux for 6 h, the solvent was removed under reduced pressure. Then, concentrated HCl (20 mL) and acetic acid (20 mL) were added for hydrolysis and the solution was refluxed overnight. After cooling, the solution was poured onto ice and aq. 40% NaOH was added until pH = 5. A pale precipitate formed. After stirring for another 30 min, the mixture was filtered, washed with water, pentane, and dried under vacuum (2.02 g, 86% yield). ^1^H NMR (400 MHz, DMSO-d_6_) δ 8.81 (d, *J* = 2.8 Hz, 1H), 8.51–8.33 (m, 2H), 7.59 (d, *J* = 9.1 Hz, 1H) Analyses were consistent with those previously reported in the literature [24].

Synthesis of 3-oxo-3*H*-benzo[f]chromene-2-carboxylic acid (CoumF)


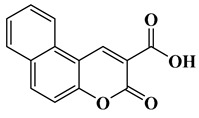


Dimethyl malonate (2.64 g, 20 mmol, M = 132.11 g/mol) and piperidine (1 mL, 10 mmol) were mixed and added to the solution of 2-hydroxy-1-naphthaldehyde (1.72 g, 10 mmol, M = 172.18 g/mol) dissolved in absolute ethanol (30 mL). After stirring and heating to reflux for 6 h, the solvent was removed under reduced pressure. Then, concentrated HCl (20 mL) and acetic acid (20 mL) were added for hydrolysis and the solution was refluxed overnight. After cooling, the solution was poured onto ice and aq. 40% NaOH was added until pH = 5. A pale precipitate formed. After stirring for another 30 min, the mixture was filtered, washed with water, pentane, and dried under vacuum (1.82 g, 76% yield). ^1^H NMR (400 MHz, DMSO-*d_6_*) δ 9.37 (s, 1H), 8.60 (d, *J* = 8.3 Hz, 1H), 8.31 (d, *J* = 9.0 Hz, 1H), 8.09 (d, *J* = 7.8 Hz, 1H), 7.77 (ddd, *J* = 8.3, 7.0, 1.3 Hz, 1H), 7.65 (dt, *J* = 12.9, 2.9 Hz, 1H), 7.60 (d, *J* = 9.1 Hz, 1H). Analyses were consistent with those previously reported in the literature [28].

Synthesis of 7-(diethylamino)-2-oxo-2*H*-chromene-3-carbaldehyde (CoumG)


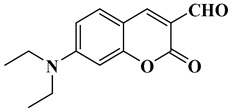


20 mL of POCl_3_ was added dropwise to 20mL of dry DMF at 0 °C under Argon and stirred during 30 minutes at 50 °C. Then 7-(diethylamino)-2H-chromen-2-one (15.0 g, 69.1 mmol, M = 217.27g/mol) in 100 mL of DMF was added to the mixture and the mixture was heated to 60 °C overnight. Afterward, the mixture was poured into 500 mL of ice water and a solution of NaOH 20% was added. The precipitate was filtered and washed with water. (13.12 g, 77% yield). ^1^H NMR (300 MHz, CDCl_3_) δ 10.08 (s, 1H), 8.21 (s, 1H), 7.46–7.32 (m, 1H), 6.61 (dd, *J* = 9.0, 2.5 Hz, 1H), 6.45 (d, *J* = 2.3 Hz, 1H), 3.46 (q, *J* = 7.1 Hz, 4H), 1.23 (t, *J* = 7.1 Hz, 6H). Analyses were consistent with those previously reported in the literature [26].

Synthesis of 5-(7-(diethylamino)-2-oxo-2*H*-chromen-3-yl)thiophene-2-carbaldehyde (CoumH)


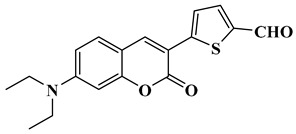


7-(Diethylamino)-3-(thiophen-2-yl)-2*H*-chromen-2-one (3.00 g, 10 mmol, M = 299.39 g/mol) was dissolved in DMF (7 mL) and POCl_3_ (1.8 mL, 20 mmol) was slowly added at 0 °C. The mixture was heated up to 80 °C overnight. After cooling, the solution was quenched with water. The mixture was extracted with DCM several times. The organic phases were combined, dried over magnesium sulfate and the solvent removed under reduced pressure. It was used without any further purification (2.88 g, 88% yield). ^1^H NMR (400 MHz, CDCl_3_) δ 9.90 (s, 1H), 8.02 (s, 1H), 7.77 (d, *J* = 4.1 Hz, 1H), 7.73 (d, *J* = 4.1 Hz, 1H), 7.38 (d, *J* = 8.9 Hz, 1H), 6.69 (dd, *J* = 8.9, 2.5 Hz, 1H), 6.56 (d, *J* = 2.4 Hz, 1H), 3.46 (q, *J* = 7.1 Hz, 4H), 1.25 (t, *J* = 7.1 Hz, 6H). Analyses were consistent with those previously reported in the literature [29].

Synthesis of 7-(diethylamino)-3-(5-(3-nitrophenyl)thiophen-2-yl)-2*H*-chromen-2-one (CoumI)


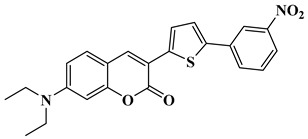


Tetra*kis*(triphenylphosphine)palladium (0) (0.46 g, 0.744 mmol, M = 1155.56 g.mol^−1^) was added to a mixture of 3-(5-bromothiophen-2-yl)-7-(diethylamino)-2*H*-chromen-2-one (2.31 g, 6.11 mmol, M = 378.28 g.mol^−1^), 3-nitrophenylboronic acid (1.53 g, 9.16 mmol, M = 166.93 g.mol^−1^), toluene (54 mL), ethanol (26 mL) and an aqueous potassium carbonate solution (2 M, 6.91 g in 25 mL water, 26 mL) under vigorous stirring. The mixture was stirred at 80 °C for 48 h under a nitrogen atmosphere. After cooling to room temperature, the reaction mixture was poured into water and extracted with ethyl acetate. The organic layer was washed with brine several times, and the solvent was then evaporated. The residue was purified by filtration on a plug of silicagel using a mixture of DCM/ethanol as the eluent (67% yield, 1.72 g). ^1^H NMR (400 MHz, CDCl_3_) δ 8.49 (t, *J* = 1.9 Hz, 1H), 8.10 (ddd, *J* = 8.2, 2.2, 1.0 Hz, 1H), 7.98–7.92 (m, 2H), 7.64 (d, *J* = 4.0 Hz, 1H), 7.56 (d, *J* = 8.0 Hz, 1H), 7.43 (d, *J* = 4.0 Hz, 1H), 7.35 (d, *J* = 8.9 Hz, 1H), 6.64 (dd, *J* = 8.8, 2.5 Hz, 1H), 6.55 (d, *J* = 2.4 Hz, 1H), 3.45 (q, *J* = 7.1 Hz, 4H), 1.24 (t, *J* = 7.1 Hz, 6H); HRMS (ESI MS) *m*/*z*: theor: 420.1144 found: 420.1147 (M^+.^ detected); Anal. calc. for C_23_H_20_N_2_O_4_S: C, 65.7, H, 4.8, O, 15.2; found: C 65.5, H 4.7, O 15.4.

### 4.2. Other Chemical Compounds

**Figure 9:**
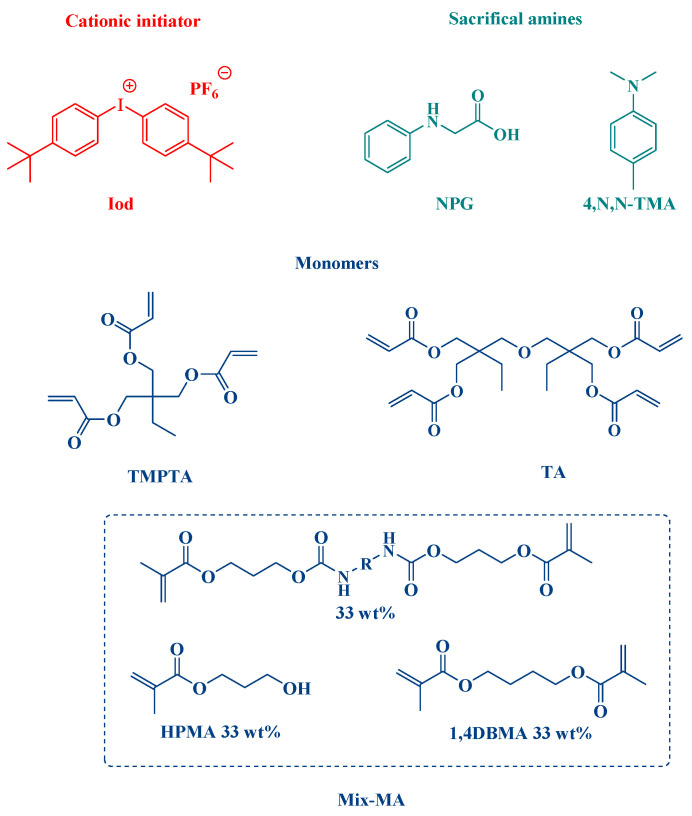
Other chemical compounds used in this paper.

All the other chemicals (Figure 9) were selected with the highest purity available and used as received. Di-*tert*-butyl-diphenyliodonium hexafluorophosphate (Iod) and TMA (4,*N*,*N*-Trimethylaniline) were obtained from Lambson Ltd. (Wetherby, UK). Trimethylolpropane triacrylate (TMPTA), di(trimethylolpropane) tetraacrylate (TA), Mix-MA, *N*-Phenylglycine (NPG) were obtained from Allnex or Sigma Aldrich (Darmstadt, Germany). TMPTA, TA, and Mix-MA were selected as benchmark monomers for the radical polymerizations.

### 4.3. Irradiation Light Sources

We used different light-emitting diodes (LEDs) as light sources: (1) at 405 nm (I = 110 mW/cm^2^) for the photopolymerization experiments, (2) at 375 nm (I = 40 mW/cm^2^) for the photolysis of Coumarins and (3) at 385 nm (I = 0.7 W/cm^2^) for the photocomposite synthesis.

### 4.4. Real-Time Fourier Transform Infrared Spectroscopy (RT-FTIR): Kinetic Followed and Final Conversion (FC) Determination for the Photopolymerisation

In this research, the ability of coumarins to initiate the photopolymerization of (meth)acrylate functions (FRP) was studied using two and three-component photoinitiating systems based on Coum/Iod salt (or NPG) (0.1%/1% *w*/*w*) and Coum/Iod/NPG (0.1%/1%/1% *w*/*w*/*w*). The percentage of the different chemical compounds is calculated according to the monomer weight. Kinetic study, as well as the reactive function conversion, were monitored by the evolution of the double bond vs. time. In fact, the polymerization experiments were performed in both thick (1.4 mm) and thin (25 µm) samples, they were obtained by deposition of the formulation into the mold (1.4 mm) or between two propylene films in order to reduce O_2_ inhibition, respectively. In addition, excellent solubility of all coumarin derivatives (excluding the CoumE) were observed. For the thick and thin samples, the evolution of the (meth)acrylate functions for TMPTA or Mix-MA were followed by RT-FTIR spectroscopy (JASCO FTIR 6600) at about 6150 cm^−1^ and 1630 cm^−1^, respectively. The procedure used to monitor the photopolymerization profile was described in detail in [30,31].

### 4.5. Redox Potentials

The reduction (E_red_) or oxidation (E_ox_) potentials for the different coumarin derivatives were determined by cyclic voltammetry in ACN using tetrabutylammonium hexafluorophosphate as the supporting electrolyte (potential vs. saturated calomel electrode–SCE). The free energy change (ΔG_et_) for an electron transfer reaction was calculated using equation (2) [27], where E_ox_, E_red_, E*, and C represent the oxidation potential of the electron donor, the reduction potential of the electron acceptor, the excited state energy level (determined from luminescence experiments) and the coulombic term for the initially formed ion pair, respectively. Here, C was neglectedm as usually done for polar solvents.

ΔG_et_ = E_ox_ – E_red_ – E* + C (2)

### 4.6. UV-Vis Absorption and Photolysis Experiments

The absorption properties (UV-visible absorption spectrum and molar extinction coefficient) as well as the steady state photolysis of the investigated Coumarin derivatives (CoumA–CoumI) in acetonitrile have been investigated using a JASCO V730 spectrometer.

### 4.7. Fluorescence Experiments

The fluorescence properties of these organic compounds in ACN were studied using a JASCO FP-6200 spectrofluorimeter. The fluorescence quenching of ^1^coumarin by Iod or NPG were examinated from the classical Stern-Volmer treatment [32] (I_0_/I = 1 + kq τ_0_[Q], where I_0_ and I stand for the fluorescent intensity of coumarin in the absence and the presence of Iod or NPG, respectively; τ_0_ stands for the lifetime of coumarin in the absence of Iod and [Q] stand for the concentration of quencher, in our study Iod or NPG).

### 4.8. Computational Procedure

Molecular orbital calculations were carried out with the Gaussian 03 suite of programs [33,34]. Electronic absorption spectra for the different compounds were calculated with the time-dependent density functional theory at the MPW1PW91-FC/6-31G* level of theory on the relaxed geometries calculated at the UB3LYP/6-31G* level of theory.

### 4.9. Near-UV Conveyor for Photocomposite Synthesis

Photocomposites have been achieved using glass fibers for the reinforcement and an organic resin based on acrylates (50%/50% *w*/*w*). The photosensitive resin has been deposited on glass fibers, then the mixture was irradiated using a LED conveyor at 385 nm (0.7 W/cm^2^). A Dymax-UV conveyor was used, the distance between the belt and the LED was fixed to 15 mm, and the belt speed was fixed to 2 m/min.

### 4.10. Laser Writing and 3D patterns Characterization

For the direct laser write experiments, a computer-controlled laser diode at 405 nm (spot size = 50 μm) was used, and the 3D patterns obtained were characterized by a numerical optical microscope (DSX-HRSU from OLYMPUS Corporation) [35].

## 5. Conclusions

Nine coumarins varying by the substitution pattern at the 3- and 7-positions of the coumarin core have been tested and proposed as highly efficient photoinitiators for the FRP of meth(acrylates) functions under visible light irradiation using a LED at 405 nm. Remarkably, these photoinitiators can be used in 3D printing experiments and these dyes showed a very high efficiency in the photocomposite synthesis (significant curing of the surface and the bottom) using a LED conveyor at 385 nm. The challenge remains, therefore, to develop new coumarins absorbing at longer wavelengths e.g., in the near-infrared range for a better penetration of light into thick/filled samples.

## Figures and Tables

**Table 1:** Light absorption properties of coumarins at 405 nm and at λ_max_; singlet state energy (E_S1_) determined from the crossing point of absorption and fluorescence spectra.

	λ_max_ (nm)	ε_max_ (M^−1^ cm^−1^)	ε_405nm_ (M^−1^ cm^−1^)	E_S1_ (eV)
CoumA	302	11,000	40	3.35
CoumB	351	13,000	80	3.24
CoumC	374	18,000	3500	3.01
CoumD	322	14,000	100	3.12
CoumE	275	18,000	60	3.91
CoumF	376	10,000	4800	2.98
CoumG	442	48,000	19,000	2.62
CoumH	458	21,000	6500	2.48
CoumI	445	27,000	1400	2.55

**Table 2:** Final reactive functions conversion (FC%) for different monomers and different PISs upon visible light irradiation using a LED at 405 nm (400 s of irradiation and thickness = 1.4 mm).

	Two-Component PISs Coum/Additives (0.1%/1% *w*/*w*)			Three-Component PISs Coum/Iod/NPG (0.1%/1%/1% *w*/*w*/*w*)		
	TMPTA	TA	Mix-MA	TMPTA	TA	Mix-MA
CoumA	n.p ^a^ 61% ^b^	n.p ^a^ 30% ^b^	n.p ^a^ n.p ^b^	80%	85%	76%
CoumB	n.p ^a^ 78% ^b^	n.p ^a^ 81% ^b^	n.p ^a^ 67% ^b^	78%	88%	76%
CoumC	60% ^a^ 42% ^b^	80% ^a^ 56% ^b^	70% ^a^ 28% ^b^	80%	88%	80%
CoumD	33% ^a^ 83% ^b^	74% ^a^ 90% ^b^	n.p ^a^ 75% ^b^	86%	92%	79%
CoumE	n.p ^a^ 36% ^b^	n.p ^a^ 25% ^b^	n.p ^a^ 30% ^b^	73%	75%	64%
CoumF	60% ^a^ 81% ^b^	86% ^a^ 88% ^b^	13% ^a^ 63% ^b^	80%	88%	64%
CoumG	70% ^a^ 75% ^b^	80% ^a^ 82% ^b^	35% ^a^ 55% ^b^	50%	40%	46%
CoumH	65% ^a^ 53% ^b^	84% ^a^ 59% ^b^	62% ^a^ 29% ^b^	81%	87%	75%
CoumI	58% ^a^ 60% ^b^	78% ^a^ 31% ^b^	24% ^a^ 18% ^b^	70%	75%	76%

^a^ Coum/Iod (0.1%/1% *w*/*w*); ^b^ Coum/NPG (0.1%/1% *w*/*w*).

**Table 3:** Final reactive functions conversion (FC%) for different monomers and different PISs upon visible light irradiation using a LED at 405 nm (150 s of irradiation and thickness = 25 µm).

	Two-Component PISs Coum/Additives (0.1%/1% *w*/*w*)			Three-Component PISs Coum/Iod/NPG (0.1%/1%/1% *w*/*w*/*w*)		
	TMPTA	TA	Mix-MA	TMPTA	TA	Mix-MA
CoumA	n.p ^a^ 32% ^b^	n.p ^a^ n.p ^b^	n.p ^a^ 22% ^b^	25%	38%	36%
CoumB	49% ^a^ 13% ^b^	32% ^a^ 24% ^b^	14% ^a^ 15% ^b^	28%	44%	52%
CoumC	25% ^a^ 25% ^b^	53% ^a^ 15% ^b^	n.p ^a^ 17% ^b^	50%	75%	72%
CoumD	42% ^a^ 34% ^b^	42% ^a^ 45% ^b^	17% ^a^ 22% ^b^	42%	70%	57%
CoumE	n.p ^a^ n.p ^b^	n.p ^a^ n.p ^b^	n.p ^a^ n.p ^b^	30%	40%	n.p
CoumF	11% ^a^ 47% ^b^	42% ^a^ 65% ^b^	18% ^a^ 70% ^b^	51%	73%	79%
CoumG	38% ^a^ 48% ^b^	68% ^a^ 67% ^b^	43% ^a^ 64% ^b^	55%	81%	74%
CoumH	19% ^a^ 14% ^b^	43% ^a^ 45% ^b^	36% ^a^ 48% ^b^	46%	68%	74%
CoumI	15% ^a^ 25% ^b^	37% ^a^ 45% ^b^	12% ^a^ 29% ^b^	42%	58%	66%

^a^ Coum/Iod (0.1%/1% *w*/*w*); ^b^ Coum/NPG (0.1%/1% *w*/*w*).

**Table 4:** Parameters characterizing the chemical mechanisms associated with ^1^Coum/Iod or ^1^Coum/NPG interaction in acetonitrile. For Iod and NPG, a reduction and oxidation potential of −0.2 and 1.03 eV were used respectively for the ΔGet calculation.

	E_ox_ (V)	E_red_ (V)	ΔG (eV) (Coum/Iod)	ΔG (eV) (Coum/NPG)	K_SV_ M^−1^ (Coum/Iod)	K_SV_ M^−1^ (Coum/NPG)	Φ (Coum/Iod)	Φ (Coum/NPG)
CoumA	-	−0.61	-	−1.71	7.25	104	0.35	0.78
CoumB	-	-	-	-	21	174	0.39	0.82
CoumC	1	-	−1.81	-	44	44	0.5	0.6
CoumD	0.6	−0.98	−2.32	−1.11	14	46.5	0.3	0.5
CoumE	-	-	-	-	5297	773	0.972	0.9
CoumF	0.39	−1.01	−2.39	−0.94	9	400	0.3	0.9
CoumG	0.46	−1.35	−1.96	−0.24	6	16	0.2	0.5
CoumH	0.96	−1.40	−1.32	−0.05	48	22	0.6	0.6
CoumI	-	−1.06	-	−0.460	4	-	0.25	-

## Data Availability

The data presented in this study are available on request from the corresponding author.

## Sample Availability

Samples of the compounds are not available from the authors.
